# Automatic lithology identification in meteorite impact craters using machine learning algorithms

**DOI:** 10.1038/s41598-024-62959-3

**Published:** 2024-06-21

**Authors:** Steven Yirenkyi, Cyril D. Boateng, Emmanuel Ahene, Sylvester K. Danuor

**Affiliations:** 1 Department of Computer Science, College of Science, Kwame Nkrumah University of Science and Technology, Kumasi, Ghana; 2 Department of Physics, College of Science, Kwame Nkrumah University of Science and Technology, Kumasi, Ghana; 3 Caburu Company Ltd, Madina, P.O. Box MD 2046, Accra, Ghana

**Keywords:** Lithology classification, Machine learning, Random forest, Bosumtwi impact crater, Space exploration, Geophysics, Computer science

## Abstract

Identifying lithologies in meteorite impact craters is an important task to unlock processes that have shaped the evolution of planetary bodies. Traditional methods for lithology identification rely on time-consuming manual analysis, which is costly and limits the efficiency of rapid decision-making. This paper utilizes different machine learning algorithms namely Random Forest, Decision Tree, K Nearest Neighbors, and Logistic Regression with Grid Search to classify rock lithologies using data from the Bosumtwi impact crater in Ghana. A repeated stratified k-fold cross-validation method is applied to Grid Search to select the best combination of hyperparameters. The findings demonstrate that the Random Forest algorithm achieves the most promising results in classifying lithologies in the meteorite impact crater with an accuracy score of 86.89%, a recall score of 84.88%, a precision score of 87.21%, and an F1 score of 85.48%. The findings also suggest that more high-quality data has the potential to further increase the accuracy scores of the machine learning algorithm. In conclusion, this study demonstrates the significant potential of machine learning techniques to revolutionize lithology identification in meteorite impact craters, thus paving the way for their influential role in future space exploration endeavors.

## Introduction

Unraveling the mysteries of meteorite impact craters requires a keen eye for lithology identification, as it provides crucial insights into the rock facies of the formations in these craters. With the help of external constraints, such as physical rock property data and borehole geophysical data, we can better understand the depth distribution of anomalous bodies and establish the existence of terrestrial impact structures. Accurately and rapidly classifying lithologies in impact craters aids in the understanding of the complex processes shaping planetary bodies, especially as space exploration becomes more established.

Previous studies have identified that lithological and structural features aid in identifying terrestrial impact craters^1–3^. Impact cratering changes the physical and lithological properties of the early planetary crust, further causing geophysical anomalies, such as increased porosity levels in allochthonous breccia deposits^1^. Thus, discerning the lithology in impact crater investigations can aid in comprehending the configuration and constitution of the rocks comprising the crater infill^2^. This information about lithology can be used to improve our understanding of impact craters' geophysical properties and the impact process. In addition, lithological information can be used to develop geological models and validate the geophysical characteristics of impact craters. Furthermore, identifying lithology from geophysical data using Machine Learning (ML) can be challenging especially due to dataset characteristics, feature count, and dataset size^4^. Reimold and Koeberl^3^ outlined the significance of lithology identification in impact craters, highlighting its potential in determining the age of impact, providing insight into the target rocks and impact event type, identifying the source of the impactor, and aiding in mineral resource exploration.

The current comprehension regarding the genesis, morphology, and mineralogy of impact craters primarily stems from geologic investigations and evaluations of drill cores from boreholes^5–8^. In the Chicxulub crater, physical property measurements on 18 core samples were used to provide a new density model that is consistent with both the 3D velocity and gravity data^5^. These measurements were utilized in calibrating the geophysical models and assigning physical properties to each lithology, which then helped in creating a well-constrained 3D structural and lithological model of the central crater^8^. In the Lappajärvi meteorite impact crater in Finland, it was noted that the intersected karnaite unit consists of three distinct layers, indicating that considerable vertical changes may have occurred within laterally homogeneous impact melt sheets^6^. In the case of the Keurusselkä structure, researchers had initially classified it as a deeply eroded impact crater lacking the typical impact lithologies, but the Vilppula drill cores revealed the presence of narrow monomictic breccia veins^7^. The optically isotropic areas in breccia thin sections were interpreted as an altered glass of either tectonic or impact origin. In the case of the Bosumtwi impact structure, the lithology identification from drill cores and physical measurements helped in developing a simple three-layer model comprising upper polymict lithic impact breccia and suevite, monomict lithic impact breccia, and basement rocks^2^.

Physical property investigations from boreholes have therefore been crucial in identifying different lithologies in the subsurface of impact craters based on mineralogical variations or changes in fabric. Traditional well-logging methods for lithology identification often struggle with complex geology (e.g., intricate sedimentary environments^9^, igneous reservoirs^10^) and rely heavily on human expert interpretations^4,11^. Human interpretations of lithology are slow, costly^4^, susceptible to depth measuring errors, inconsistent^11^ and, subjective. There is therefore an urgent need for an innovative solution that can address the challenges associated with lithology determination through physical response measurements from boreholes. The solution should be cost-effective and free from subjective factors while providing accurate lithology mapping. This is especially true in the context of the increased need to understand the lithology of other planetary bodies^12,13^ in our solar system in the age of accelerated space exploration. Machine Learning Algorithms (MLAs), when provided with extensive datasets, excel in extracting complex and non-linear relationships between physical properties and lithology, thereby enhancing the precision of lithology detection in certain samples. It is an efficient alternative that analyzes vast amounts of well-logging data and identifies subtle patterns that may be missed by humans. This data-driven approach can lead to more accurate and objective lithology identification, reduced dependence on subjective expert interpretation, and faster analysis times compared to traditional methods.

MLAs such as Support Vector Machines (SVM)^14^, Decision Trees (DT)^4,15^, Random Forest (RF)^14,16^, XGBoost^17,18^, Logistic Regression (LR)^19^, K Nearest Neighbors (KNN)^20,21^ and Artificial Neural Networks^4,22^ have proven effective for lithology identification, especially in petroleum exploration. However, a challenge remains due to the lack of quality training data^23^, which can negatively impact prediction performance^24^. In this study, we propose a data-driven methodology for predicting lithologies in a meteorite impact crater using physical measurements from drill cores. The five lithologies considered are Metagreywacke (MGW), Shale Slate Phyllite Schist (SSP), Monomict Lithic Breccia (MLB), Polymict Lithic Breccia (PLB), and Suevite (SUE). Our approach involves fine-tuning MLA hyperparameters and selecting the best combination for lithology prediction. To our knowledge, this is the first application of MLAs for lithology prediction in a meteorite impact crater using physical drill core data.

## Results

The primary goal of this study is to evaluate the efficacy of MLAs in identifying lithologies in impact craters. Four MLAs were employed, namely KNN, LR, DT, and RF. The subsequent sections provide a comprehensive analysis of the outcomes obtained from these models. Our investigation begins by finding the best set of parameters for each ML model. Subsequently, the investigation proceeds with an examination of the confusion matrices generated by each ML model. To provide a visual representation of the model predictions, well-log plots are presented, featuring the original lithology plot alongside the predicted lithology plot. The evaluation process extends to encompass overall accuracy, F1 score, and precision scores for each ML model.

### Confusion Matrices

**Figure 1:**
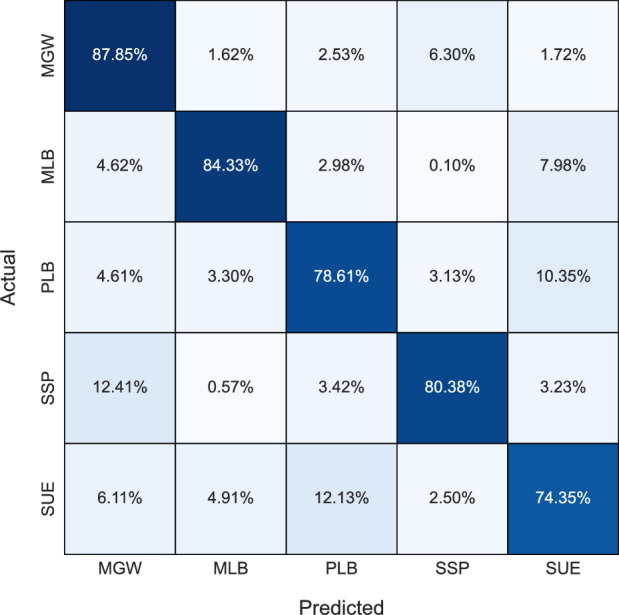
Confusion matrix for the Decision Tree model.

**Figure 2:**
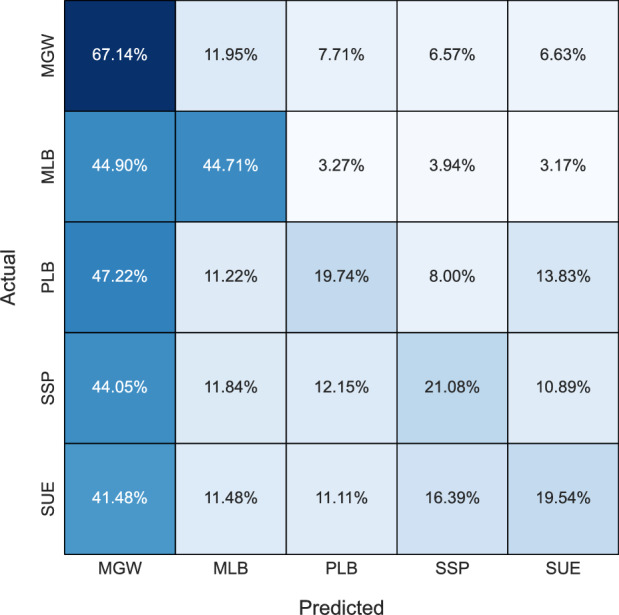
Confusion matrix for the K Nearest Neighbor model.

**Figure 3:**
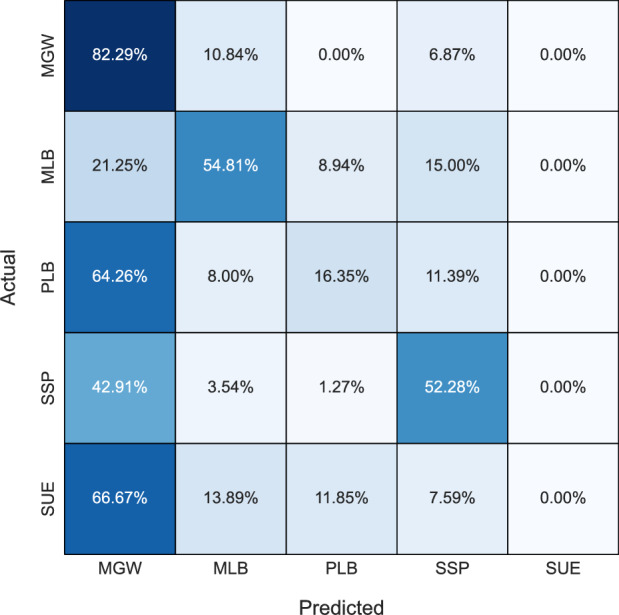
Confusion matrix for the Logistic Regression model.

**Figure 4:**
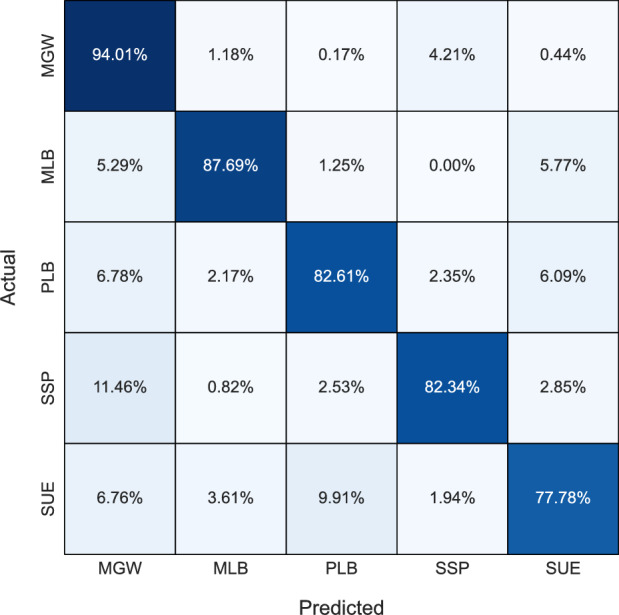
Confusion matrix for the Random Forest model.

The evaluation of lithology identification by ML models begins with the evaluation of the confusion matrices yielded. The confusion matrices for DT, KNN, LR, and, RF are presented in Figs. 1, 2, 3, and 4 respectively. A confusion matrix visualizes the lithology-specific classification performance of an ML model. There are four kinds of classification outcomes in a confusion matrix: True Positive and True Negative, denoting instances where the model accurately predicts the lithology of a sample; and False Positive and False Negative, representing cases where the model misclassifies a given sample. The diagonal of the matrix shows the percentage of correctly classified lithology classes.

In general, KNN and LR tended to exhibit bias towards the MGW class, possibly stemming from the algorithms’ tendency to overfit the dataset, which notably contains a higher proportion of MGW samples compared to other classes. Consequently, these models prioritized the MGW class during classification, resulting in imbalanced predictions across different classes. The bias towards MGW in KNN can be further explained by its classification process, which simply selects the majority class of the k nearest neighbors surrounding a given input. Hence, due to the greater number of MGW samples, it may often be the majority class surrounding any given input. Apart from RF, no other model achieves a classification rate above 90% for any lithology. Despite SUE occurring only 4 times more than MLB (104 vs. 108) in the dataset, all models struggle to classify SUE, achieving a maximum accuracy score of 77.78%, whereas MLB achieves accuracies surpassing 84% with tree-based models. This underscores the inherent complexity of the SUE lithology. Overall, tree-based models exhibit superior lithology classification accuracy and demonstrate their robustness to class imbalance.

### Evaluation metrics scores

Table 1 displays the performance metrics of the classification models evaluated in our study while Table 2 presents the optimized hyperparameter values. Each model's accuracy, recall, precision, and F1 score are reported, providing insights into their classification capabilities. RF emerges as the top performer, achieving an overall accuracy of 86.89% and demonstrating robust recall, precision, and F1 scores above 84%. DT also exhibits commendable performance, with an accuracy of 82.64% and well-balanced recall, precision, and F1 scores exceeding 80%. However, the performance of KNN and LR models falls notably short, with accuracy scores of 41.31% and 51.51% respectively. These models show lower recall, precision, and F1 scores, indicating significant challenges in accurately classifying the data.

**Table 1:** Overall accuracy, recall, precision, and F1 scores were obtained from a repeated 15-fold stratified cross-validation using four classifiers.

Model	Accuracy (%)	Recall (%)	Precision (%)	F1 (%)
DT	82.64	81.10	81.87	80.93
RF	86.89	84.88	87.21	85.48
KNN	41.31	34.43	36.52	33.59
LR	51.51	41.14	41.21	41.21

**Table 2:** Optimized hyperparameters for machine learning algorithms using grid search cross-validation.

Algorithms	Tuned hyperparameters
DT	Criterion = gini Max depth = none Max features = none Min samples leaf = 1 Min samples split = 2 splitter = best
RF	Criterion = gini Max depth = none Max features = none Min samples leaf = 1 Min samples split = 2 n estimators = 50
KNN	Algorithm = brute n neighbors = 3 Weights = none
LR	C = 0.1 Max iter = 100 Penalty = l2

## Discussion

In this study, we evaluated the performance of four ML models—KNN, RF, DT, and LR—in identifying lithologies. Our analysis revealed notable differences in the classification accuracy of these models, with some displaying higher proficiency than others.

While analyzing the data, a consistent pattern of high accuracy in classifying the MGW lithology across all models was observed, which aligns with expectations. This observation is noteworthy, particularly given that the MGW lithology constitutes a significant proportion (38%) of the dataset, while none of the other four lithologies represent more than 20% of the data. We posit that this class imbalance may have influenced the models' bias towards the majority class (MGW), leading to a tendency to classify other lithologies as MGW.

The implications of class imbalance on model performance are noteworthy. Class imbalance can introduce bias in ML models, where they prioritize the majority class while struggling with minority classes. This phenomenon was evident in our study, where the KNN and LR models tended to favor the MGW lithology, potentially at the expense of accurate classification of other lithologies.

However, it's important to note some limitations. Firstly, our analysis was based on a specific dataset, potentially limiting the generalizability of our findings. Additionally, due to constraints in data access, we had to extract information from graphical images, which might have introduced variability in data quality, possibly affecting the performance of our ML models.

KNN has a simple implementation allowing it to have a short training time, however, its performance hinges on the choice of the “k” parameter^25^. KNN assigns the majority class in the k-neighborhood to a given input. This majority voting-based classification rule degrades KNN’s performance on class-imbalanced datasets^26^, such as the one used in this study. Non-linear data, such as lithology data, pose significant challenges for the LR model. Linear models like LR lack the flexibility to capture the complexity of non-linear data^27^ and are prone to overfitting to noise when faced with non-linear data relationships^28^. DT is robust against class imbalance and effectively captures the non-linear characteristics^29^ of lithology data. Nevertheless, DT has a general tendency to overfit the training data^30^, limiting its classification performance in the process.

In the domain of lithology identification, the RF algorithm stands out as the preferred choice due to its superior performance across key metrics. The ensemble nature of RF, constructed from multiple trees based on random subsets of features and data points, safeguards against overfitting, and enables the algorithm to navigate the complexities of lithology identification adeptly. Notably, RF excels in handling imbalanced datasets, providing a natural balance through random sampling with replacement during the bagging process. This innate capability ensures accurate identification of even minority lithologies, a strength absent in many other models. The grid search optimization further refined RF's performance, with parameters such as the gini impurity criterion and an ensemble size of 50 trees proving optimal. In summary, RF's ensemble strength, feature importance analysis, robustness in handling imbalanced data, and fine-tuned parameters collectively position it as a powerful tool for accurate and insightful lithology identification.

The findings presented in this study support the applicability of tree-based algorithms for lithology classification, aligning with earlier research^14,31,32^ that identifies RF as a favorable ML algorithm for this task. This convergence of evidence further reinforces RF's superior performance in the accurate classification of lithologies.

We further delve into the visual exploration of lithology predictions using well-log plots, focusing exclusively on the predictions generated by the RF model—the top-performing ML model identified in previous analyses. These well-log plots juxtapose the actual lithology data with the predicted lithology data, offering a comprehensive visual representation of the RF model's efficacy in lithology identification within impact craters. Confronted with the challenges posed by small and imbalanced datasets, a pragmatic strategy was adopted. Before merging the well-logs, an identifier was added to each sample, simplifying the subsequent reconstruction of the two well-logs datasets post-prediction.

**Figure 5:**
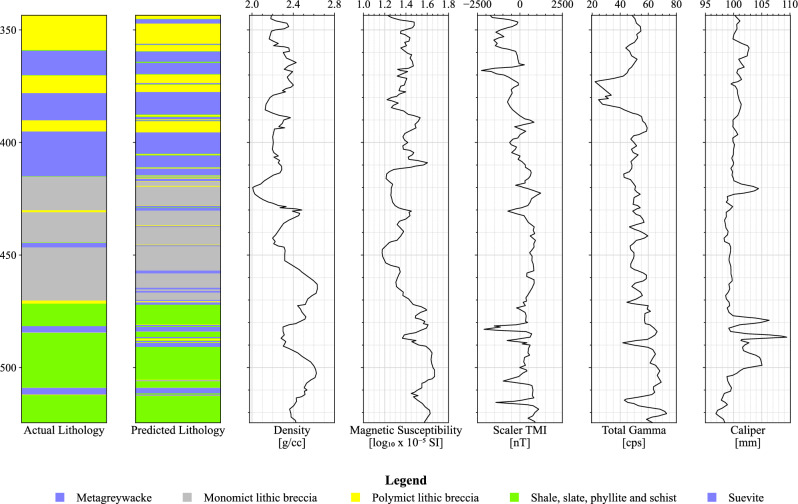
Lithology predictions generated by the Random Forest model for well-log LB-07A.

**Figure 6:**
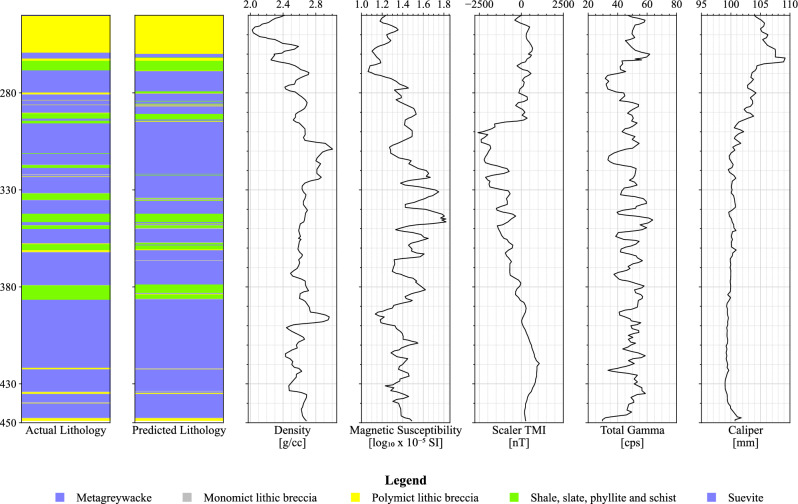
Lithology predictions generated by the Random Forest model for well-log LB-07A.

The RF’s model performance was assessed through a repeated stratified k-fold cross-validation approach, ensuring a robust evaluation of its lithology identification capabilities. The predictions, coupled with their cross-validation iteration, were saved during this process. By pinpointing the iteration with the highest overall accuracy score, we identified the most effective model performance. Subsequently, only the data from this optimal iteration were separated back into the individual well logs. From these specifically optimized predictions, we present and analyze the lithology plots. Figures 5 and 6 showcase the well-logs featuring predictions made by the RF model for LB-07A and LB-08A, respectively.

One significant challenge faced is limited data. Scientific drilling projects, such as ICDP that generated our datasets involve substantial investment due to equipment, personnel, and logistical requirements. This economic reality requires strategically choosing drilling locations, resulting in a smaller number of boreholes compared to an ideal uniform grid pattern. Thus, the dataset sourced from results from the ICDP project reflects this limitation, as evidenced by the severely imbalanced and limited nature of the datasets. This data limitation is addressed using techniques employed in this study. For instance, stratified k-fold cross-validation was specifically chosen to account for the imbalanced class distribution within the combined dataset. Despite these real-world limitations in data acquisition, the techniques employed in this study demonstrate the potential of ML for lithology classification in impact craters. This is particularly relevant in space exploration endeavors, where missions to celestial bodies like Mars often involve rovers with limited drilling capabilities.

Lithological identification is important as a constraint on other forms of data used in impact crater studies. For instance, even though geophysical data such as magnetic data can often suggest hidden impact craters, they are not non-ambiguous. Therefore, understanding the distribution of lithologies is important as a constraint through either physical property data from the study area or borehole geophysical data. Borehole geophysical data provides constraints on the depth distribution of depth bodies and the logs can be used to identify lithologies rapidly and therefore avoid the expensive coring required in each borehole drilled. Therefore, the machine learning approach can be useful in easily predicting lithology.

## Methods

### Workflow overview

**Figure 7:**
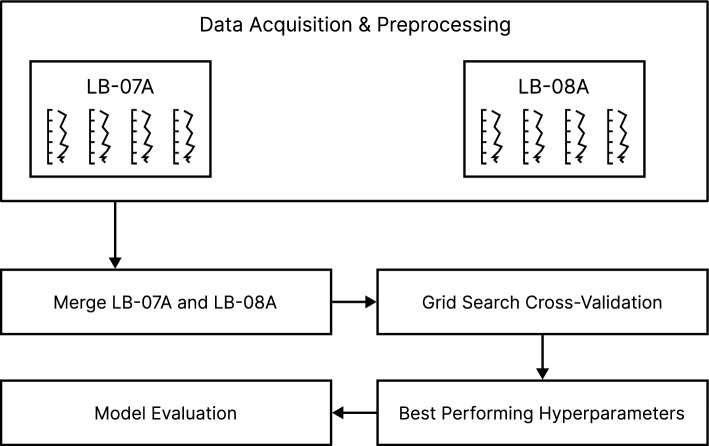
Workflow for the proposed machine learning process.

Figure 7 displays the schematic of the proposed ML workflow, which is mainly composed of the following parts: data acquisition and preprocessing, hyperparameter tuning using Grid Search (GS), and evaluation of the tuned model. A detailed description of each part will be treated in subsequent sections. The principal interest of this work is to assess the ability of MLAs to predict the lithologies found in a meteorite impact crater.

### Data acquisition and preprocessing

The application of MLAs in predicting lithologies was investigated through the analysis of two boreholes, LB-07A and LB-08A, drilled within the Bosumtwi Impact Crater as part of the International Continental Scientific Drilling Program (ICDP)^2^. Physical property measurements and borehole log information were obtained, including density and magnetic susceptibility measurements on the core material, and borehole total magnetic field data extracted from the borehole deviation surveys^33^. These measurements, along with total gamma radiation and caliper data, were presented as borehole logs^2^.

**Figure 8:**
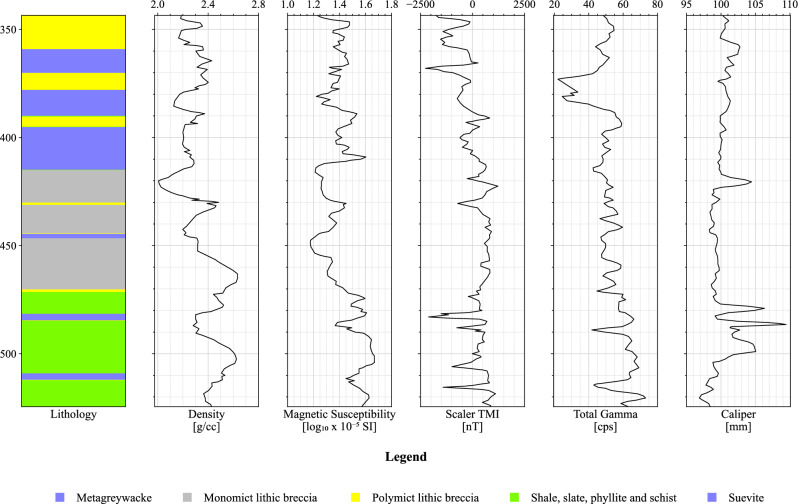
Physical property logs for borehole LB-07A (digitized and extracted from^2^).

**Figure 9:**
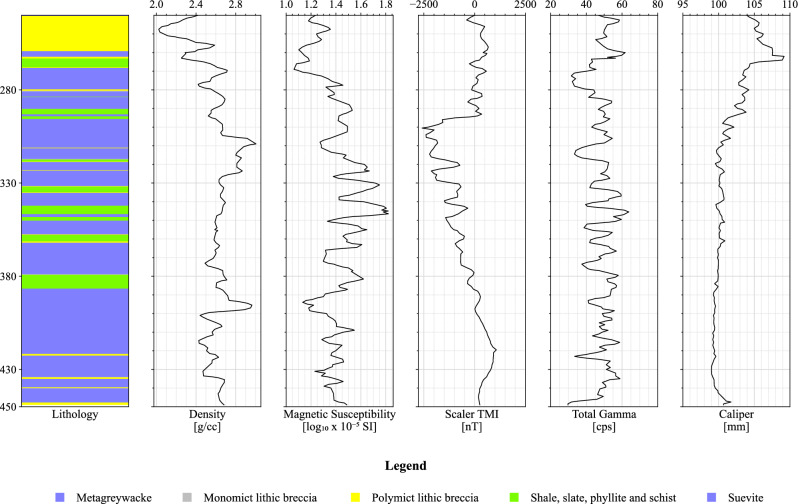
Physical property logs for borehole LB-08A (digitized and extracted from^2^).

Due to the inaccessibility of the original data, the borehole logs were extracted from figures in Morris et al.^2^ using a digital tool for this purpose^34^. The final output was then plotted and visually compared with the original borehole plot from Morris et al.^2^ to ensure fidelity to the original plots. Figures 8 and 9 show the petrophysical property logs for boreholes LB-07A and LB-08A, respectively. Both boreholes have the following individual logs: (a) simplified lithology; (b) density (g/cm^3^) measured on core segments; (c) magnetic susceptibility ($\text{log}10\times {10}^{-5}$ SI) measured on core segments; (d) Total Magnetic Intensity (TMI) (nT), scalar magnetic intensity derived from borehole deviation survey; (e) total gamma derived from borehole survey by ICDP (cps); and (f) caliper (mm) survey.

The dataset includes two wells with five features each: density, total gamma, caliper, scaler TMI, and magnetic susceptibility. Five lithology classes are targets for classification. These targets are MGW, SSP, MLB, PLB, and SUE. Table 3 shows the number of samples for each lithology in LB-07A and LB-08A datasets after preprocessing.

**Table 3:** Number of samples per lithology for the LB-07A and LB-08A datasets.

Lithology	LB-07A	LB-08A	Total
Metagreywacke (MGW)	12	285	297
Shale Slate Phyllite Schist (SSP)	94	64	158
Monomict Lithic Breccia (MLB)	104	0	104
Polymict Lithic Breccia (PLB)	63	52	115
Suevite (SUE)	90	18	108
Total	363	419	782

In the preprocessing stage, several key aspects were addressed to ensure the integrity and suitability of the well-log data for subsequent ML analysis. The two well-log datasets, characterized by small size and high-class imbalance, were combined. This was done to increase the overall dataset size and enhance the model’s ability to discern lithologic patterns. Before merging, a new dataset identifier feature was introduced for each sample to facilitate post-prediction reconstruction of the two well-log datasets. To maintain data quality, samples with missing values for any of the features were systematically removed. Finally, the lithology target variable was encoded, mapping classes to numeric values for compatibility with algorithms.

### Hyperparameter optimization

Choosing an appropriate MLA for a given task is one step in developing an effective ML model. The other step is to obtain an optimal architecture for the algorithm by tuning its hyperparameters. Hyperparameter tuning is the process of finding the set of hyperparameters that produce the most effective ML model for a given task. Hyperparameters are parameters whose values must be specified before the learning process^35^ and the selection of the appropriate values is done by the ML practitioner or through an optimization process. GS is a hyperparameter optimization approach that exhaustively searches for the optimal combination of hyperparameters from a fixed domain of hyperparameters^36^. It involves specifying a range of values for selected hyperparameters, after which the performance of the MLA is thoroughly assessed for every combination of these hyperparameters. The following steps outline the GS hyperparameter tuning process.

1. Define a set of hyperparameters $H$ and a range of values for each. Let ${h}_{i}$ represent a specific hyperparameter, ${h}_{i}^{1},{h}_{i}^{2},\ldots ,{h}_{i}^{{k}_{i}}$ represent the range of values for the hyperparameter ${h}_{i}$, and ${k}_{i}$ represent the number of values for ${h}_{i}.$
2. Construct a grid containing all values of combinations of hyperparameter values. The grid represents the entire search space. For $n$ parameters, the total number of grid points is ${k}_{1}*{k}_{2}*\dots *{k}_{n}$. Each grid point is denoted as $p$.
3. For each $p$, set the MLA’s hyperparameter values, then train the algorithm on the training dataset and evaluate it on the test dataset using repeated stratified k-fold cross-validation to obtain the accuracy of the model.
4. Select the combination of hyperparameters that produce the best performance using the evaluation metric.

GS is intuitive and easily implemented. Nevertheless, as the hyperparameter space expands with the inclusion of more hyperparameters and values, GS encounters a challenge known as the curse of dimensionality^36^. This results in an increase in the number of grid points for evaluation.

### Description of machine-learning algorithms

Four MLAs were used in this study: Decision Tree (DT), Random Forest (RF), Logistic Regression (LR), and K Nearest Neighbors (KNN). These four MLAs were selected for their well-established nature, easy understanding of their decision-making process, and extensive application across various geophysical tasks. This section provides a description of each of the four ML models and an overview of their hyperparameters.

**Figure 10:**
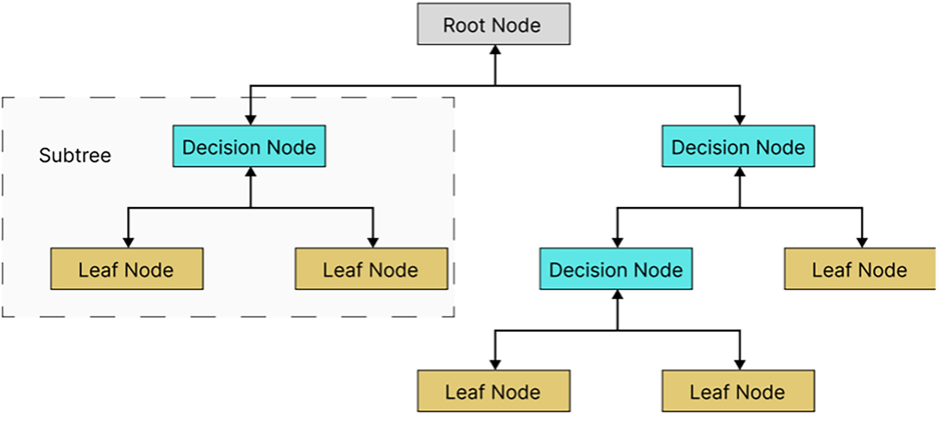
Decision Tree structure.

The structure of a DT is presented in Fig. 10. The DT algorithm is a tree-structured classifier in which features are represented as internal or decision nodes, decision rules are represented as branches, and results are represented as leaf nodes. Branches (or decision rules) represent the chance outcomes that emanate from the root node, and they are used to create the hierarchy of the tree. DT is a powerful algorithm used in various lithology classification tasks^4,37,38^. A DT can be regarded as a deterministic algorithm for deciding which variable to test next, based on the previously tested variable and the results of their evaluation until the function's value can be determined. Fig. 4 illustrates the structure of a DT. Six DT hyperparameters were optimized in this study: criterion (assess split quality), splitter (chooses the best splitting strategy: random or best), max depth (prevents overfitting by limiting tree depth), min samples split (determines the number of data points required to split a decision node), min sample leaf (sets minimum samples per leaf node), and max features (reduces overfitting by limiting features considered for splits)

**Figure 11:**
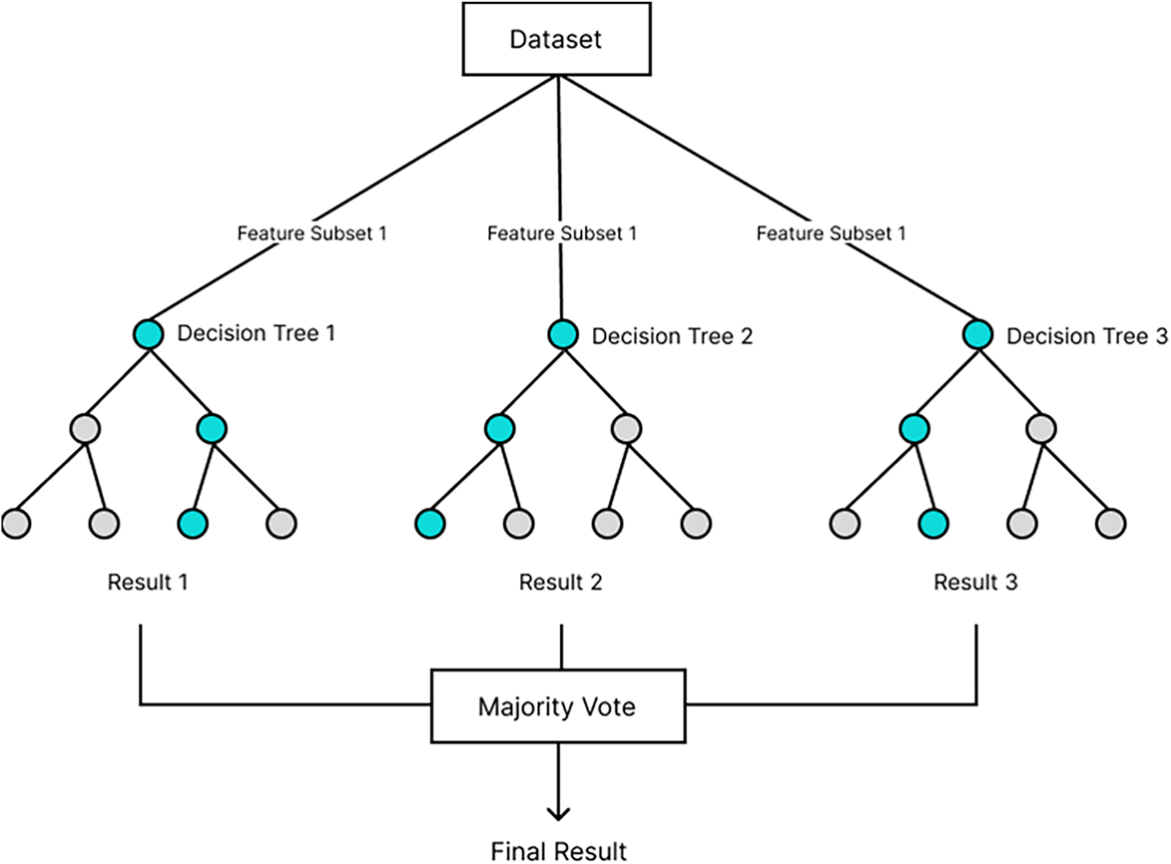
A schematic illustration of a random forest algorithm.

RF is an ensemble ML algorithm that uses a group of DTs. Each tree is dependent on a random vector that is sampled independently and with the same distribution for all trees in the forest^39^. As the number of trees increases, the generalization error for the RF algorithm converges almost surely to a limit. The generalization error of RF classifiers is influenced by the strength of the individual trees and the correlation between them. Figure 11 shows a schematic illustration of the RF algorithm. The main idea behind RF is to use multiple uncorrelated DT models to predict a label for each instance. Unlike in DT, where the best feature is selected out of all the features, and where the entire dataset is used, trees in RF do not get access to all features and data. RF uses feature randomness or bagging to ensure each DT is not correlated to other DTs. With feature randomness, each tree selects the best feature out of a random subset of features. The bagging method ensures each tree is trained on a different random sample of the training data. RF is effective because it is a highly versatile and effective MLA that delivers precise predictions across diverse applications, while also enabling feature importance assessment during model training and facilitating the computation of pairwise proximity between samples^40^. RF has been applied in several areas such as bioinformatics^40^, subsidence susceptibility assessment^41^, lithology classification^4^, fault detection^42^, and facies and fracture prediction^43^. Six RF hyperparameters were optimized in this study: criterion (assess split quality), n estimators (specifies the number of DTs in the forest), max depth (prevents overfitting by limiting tree depth), min samples split (determines the number of data points required to split a decision node), min sample leaf (sets minimum samples per leaf node), and max features (reduces overfitting by limiting features considered for splits)

LR is an extension of the linear regression method. While linear regression models continuous outcomes and assumes a linear relationship between the outcome and independent variables, logistic regression is used for binary classification tasks, predicting the probability of a certain outcome occurring^44^. In multi-class prediction tasks, such as identifying different lithologies, LR is adapted to handle multiple classes. Some of the adaptations include the one-vs-rest/one-vs-all^45^ approach where separate LR models are trained for each class and the final classification of an input is the LR model with the highest probability. Another approach is the multinomial (SoftMax) LR where a single LR model with multiple output classes is trained simultaneously. LR estimates the conditional probability, denoted as $Pr\left(G|X\right)$ where $G$ represents the target class and $X$ represents the input features. One advantage of using LR is its interpretability which is a desired feature in many ML applications. Three LR hyperparameters were considered in this study: penalty, C, and max iter. The penalty hyperparameter specifies the type of regularization (l1, l2, elasticnet, or None) to help prevent overfitting. C controls the trade-off between fitting the training data and generalization. Max iter sets the maximum number of iterations for the solvers to converge.

**Figure 12:**
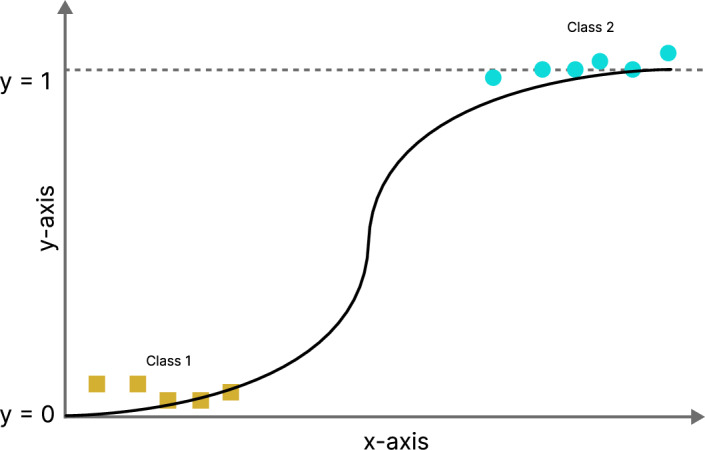
Logistic regression graph.

The graph of a logistic function (Fig. 12) represents the relationship between the input and the probability of the input belonging to a certain class. The logistic function converts the linear combination of input variables into a probability between 0 and 1. The x-axis of the graph represents the values of the input variables. In contrast, the y-axis represents the predicted probability of the outcome variable being in one of the classes. The x-axis of the graph represents the values of the input variables. In contrast, the y-axis represents the predicted probability of the outcome variable being in one of the classes.

The KNN algorithm is quite straightforward: for a given input, its class is determined by the majority class among its k nearest neighbors, where k is a positive number. In regression with KNN, the value of a given input is simply the average of its k nearest neighbors. The distance from an input to its k nearest neighbors is typically measured using the Euclidean distance. KNN is non-parametric and lazy^25^, meaning it doesn't assume data distribution or require training before predictions. It memorizes the training dataset and predicts based on local patterns. However, its performance can depend on k and the distance metric^25^, and it may be computationally complex for large datasets.

**Figure 13:**
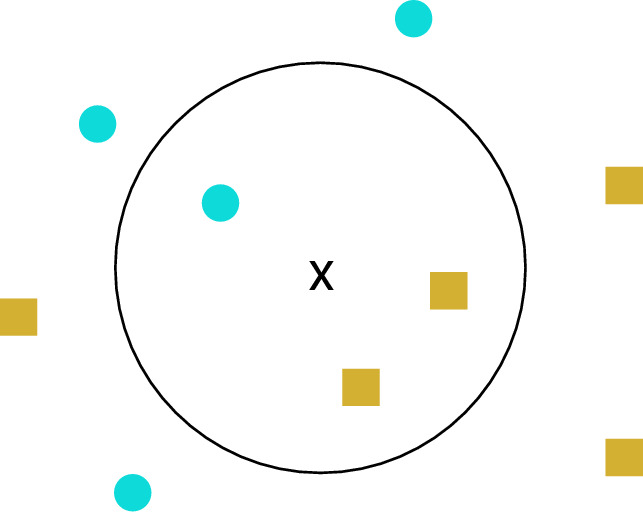
KNN feature space.

Figure 13 illustrates the feature space of the KNN algorithm. The circular boundary contains training data inputs closest to the x input awaiting prediction. The x input will be classified as a square because, among the three closest shapes, two are squares. Three KNN hyperparameters were optimized in this study: k, weights, and algorithm. The k hyperparameter determines the number of neighbors to consider when making predictions. The weights hyperparameter determines the weight given to each neighbor when making predictions. It can be set to either ‘uniform’, where all neighbors are weighted equally, or ‘distance’, where closer neighbors have more influence on the prediction. Finally, the algorithm hyperparameter specifies the algorithm used to compute the nearest neighbors.

### Training and evaluation

The computational experiments presented in this paper were conducted in Python with the sci-kit-learn library. Given the constraints of limited and imbalanced well-log datasets, it was impractical to employ one well-log for training and another for evaluating the trained model. Instead, the LB-07A and LB-08A well-logs were combined to enhance the robustness of the ML-based lithology identification models. This amalgamation served a dual purpose: it increased the overall dataset size and improved the generalizability of MLAs by exposing them to a more comprehensive range of lithologies. Subsequently, each of the MLA’s hyperparameters was optimized using GS combined with a repeated stratified k-fold cross-validation. The average overall accuracy, recall, precision, and F1 scores of the optimized ML models were obtained for evaluation, as well as their confusion matrices for further lithology-specific evaluation. Finally, the lithology predictions of the best-performing model were plotted alongside the actual lithology. Inferior quality and class imbalance in LB-07A and LB-08A logs rendered traditional evaluation methods impractical. To overcome this, the predictions for each well-log were plotted by first saving the predictions from the repeated stratified k-fold cross-validation on the combined dataset and subsequently reconstructing the LB-07A and LB-08A from these saved predictions.

## Conclusion

This study proposes a workflow based on MLAs to automatically identify lithology in an impact crater using physical measurements from the core and logging data. The workflow effectively tackles real-world challenges like high-class imbalance and limited data availability, demonstrating that MLAs can achieve optimal performance despite these constraints.

The methodology was applied to field data acquired at the Bosumtwi Impact Crater in Ghana during the Lake Bosumtwi Drilling Project (BCDP) 2004. The workflow produced models with good classification accuracies, which can help geoscientists determine lithologies in impact craters and expand our knowledge in this area. The hyperparameters of the MLAs were tuned using GS and a repeated stratified 10-fold cross-validation technique is used to assess and compare the performance of the MLAs. Notably, the RF algorithm attained an accuracy score of 86.89%, a recall score of 84.88%, a precision score of 87.21%, and an F1 score of 85.48%.

In the era of space exploration, planetary geophysicists can leverage this workflow to enhance the accuracy of geological mapping, diminishing the reliance on manual analysis and enabling real-time lithological decision-making. These advancements aim to deepen our comprehension of planetary geology.

## Data Availability

The dataset utilized in this study was sourced from images published in a peer-reviewed paper by Morris et al.^2^. These datasets, extracted for analysis, are readily accessible to the public via the following link: https://github.com/stevenyirenkyi/bosumtwi.

## Code availability

The tuned machine learning models and the extracted datasets are publicly available at https://github.com/stevenyirenkyi/bosumtwi.
